# The Cholera in Germany

**Published:** 1860-03

**Authors:** 


*The Cholera in Germany.*—A letter from the Grand Duchy of Mecklenbnrg, in the *Augsburg Gazette*, says: The cholera has broken out with great violence in some towns and villages of this duchy, and many of the persons attacked have died after four or five hours' suffering. In some of the villages the harvest operations have been suspended for want of hands, sixty to seventy persons having been taken ill at the same time. The disease breaks out first in one place and then in another, sparing for a time intermediate villages, and then turning back on them with increased violence. The ports of Rostock and Warnemunde have not escaped the malady which was brought there, it is supposed, by a vessel from St. Petersburg. The cholera continues to rage at Hamburg, carrying off from sixty to seventy persons daily.—*Virginia Medical Journal.*

